# Plant-based diet and COVID-19 severity: results from a cross-sectional study

**DOI:** 10.1136/bmjnph-2023-000688

**Published:** 2023-10-04

**Authors:** Samira Soltanieh, Marieh Salavatizadeh, Tooba Ghazanfari, Soodeh Razeghi Jahromi, Zahra Yari, Mohammad Ali Mansournia, Maryam Nazemipour, Jalil Arab Kheradmand, Sussan K Ardestani, Sara Karimi, Azita Hekmatdoost

**Affiliations:** 1 Department of Clinical Nutrition and Dietetics, Shahid Beheshti University of Medical Sciences, Tehran, Iran (the Islamic Republic of); 2 Immunoregulation Research Center, Shahed University, Tehran, Iran (the Islamic Republic of); 3 Department of Epidemiology and Biostatistics, Tehran University of Medical Sciences, Tehran, Iran (the Islamic Republic of); 4 Ahya Neuroscience Research Center, Tehran, Iran (the Islamic Republic of); 5 Department of Immunology, University of Tehran, Tehran, Iran (the Islamic Republic of)

**Keywords:** COVID-19, Dietary patterns

## Abstract

Although previous findings have shown the beneficial role of healthy eating pattern on the human immune system, the association between plant-based diet and COVID-19 severity has not yet been elucidated. This study aimed to determine the possible role of plant-based diet index (PDI) in COVID-19 severity. This cross-sectional, multicentral study was conducted on 141 patients with confirmed COVID-19. Dietary intakes of the patients were evaluated using a validated food frequency questionnaire. Then, PDI was compared between patients who needed to be hospitalised (considered severe cases), and those who got treatment at home (considered non-severe cases). After adjustment for confounders including age, sex, energy intake and body mass index, lower odds of hospitalisation were found for participants having a greater score of overall PDI (OR per 10 units increase: 0.42; 95% CI 0.22 to 0.80) and healthy PDI (OR per 10 unit increase: 0.45; 95% CI 0.26 to 0.78). In conclusion, our data presented that there is a relation between PDI and lower risk of hospitalisation in COVID-19 patients, possibly through boosting the immune function.

**WHAT IS ALREADY KNOWN ON THIS TOPIC**

- The beneficial role of healthy eating pattern on the human immune system has been shown previously.

**WHAT THIS STUDY ADDS**

- Our data presented that there is a relation between plant-based diet index and lower risk of hospitalisation in COVID-19 patients, possibly through boosting the immune function.

**HOW THIS STUDY MIGHT AFFECT RESEARCH, PRACTICE OR POLICY**

- These findings indicate that following a plant-based diet can ameliorate COVID-19 disease symptoms and signs.

## Introduction

COVID-19 pandemic has exerted an outsize influence on people’s lifestyles, food choices, food security and food access in different groups of society all over the world.^1^ Nutritional factors are known as a key element affecting immunity, risk of infection and mortality. The impact of adaptable risk factors such as diet, exercise, healthy lifestyle on regulation of inflammatory mediators to lower the risk of infection is well documented.^2^ On the contrary, leading unhealthy lifestyle (including exacerbating dietary patterns and inactivity) is considered to be linked with an increased level of oxidative stress being conducive to progression of non-communicable diseases.^3 4^ Experimental researches and studies lend evidence to the fact that a myriad of vitamins (A, B_6_, B_12_, folate, C, D and E) and trace elements (zinc, copper, selenium, iron) play a crucial role in supporting immune system against bacteria and viruses.^5^ Taking healthy eating measures which is consistent with consumption of a diverse diet, various plant-based and animal-based foods have a profound impact on boosting immune defence.^6^ Many infectious diseases stem from gut dysbiosis that results in an uncontrolled excessive inflammatory responses that can worsen COVID-19.^7^ Healthy dietary patterns, many plant foods, fibres and fermented foods can modify and maintain gut microbiota for further immune supply against infections, including of the respiratory tract.^6^ Kim *et al* conducted a case–control study on 568 COVID-19 cases (138 moderate-to-severe cases, 430 very mild to mild COVID-19 severity) and 2316 controls. Their findings reported that individuals who followed ‘plant-based diets’ had 73% (OR 0.27, 95% CI 0.10 to 0.81) lower odds of moderate-to-severe COVID-19 severity as opposed to participants who did not follow this diet.^8^ Plant-based diets are rich in whole grains, legumes, vegetables, potato, fruits, seeds and low in meat consumption.^9^ The plant foods are great sources of protease inhibitors like trypsin and trypsin-chymotrypsin inhibitors, which are bioavailable and are able to prevent SARS-CoV-2 from binding to the host cells.^10^

To the best of our knowledge, there is no published study regarding the association of plant-based diet index (PDI) and COVID-19 severity. The purpose of this study is to investigate whether plant-based diets using PDI might be useful to alleviate COVID-19 severity and prevent patients from getting hospitalised. Although clinical studies on this matter are scarce, there is evidence from previous prepandemic epidemiological, observational and clinical studies that demonstrates the benefits of plant-based diets on some conditions that can also be found in individuals with COVID-19.

## Materials and methods

### Study population

This cross-sectional study was conducted on 141 individuals (75 men, 66 women) with the mean age of 46.23 years. Inpatient participants were collected from Imam Khomeini hospital, Tehran, Iran, and outpatients were recruited from Simorgh clinical laboratory. The details of sampling were presented previously.^11^ Individuals with ascertained COVID-19 infection by a specialist were opted for the research. Participants with history of diabetes, hypertension, kidney disease, cancer, and cardiovascular diseases, consuming nutritional supplements, and/or some special diets in the last year, any alternations in the dose and type of one’s medicine, pregnancy and lactation, and unwilling or unable to cooperate were excluded from the study. Informed consent was obtained from all participants. People who met the criteria were divided into two groups: (1) severe cases included patients who needed hospitalisation except for the ICU unit, required non-invasive breathing support or administration of antibiotics combined with oxygen support and (2) non-severe cases included patients treated as outpatients in what is mentioned as home-hospitalisation. Demographic, clinical and laboratory data were included from the study entitled ‘Immunological aspects of COVID-19 in selected provinces of Iran’,^11^ approved by ‘National institute for medical research development’ (IR.NIMAD.REC.1399.041).

### Dietary assessment

Assessment of dietary intakes of participants was done using a valid and reliable semiquantitative food frequency questionnaire containing 147 food items.^12^ Individuals were asked to report their frequency of intake of each food item during the past year daily, weekly or monthly by Trained researchers via telephone interview. The reported portion sizes were converted to daily intakes. Using Nutritionist IV software (V.7.0; N-Squared Computing) adapted for Iranian foods, the nutrient content of each food item was calculated.

### Plant-based diet indices

Total PDI and healthful PDI (hPDI) were calculated by means of previous methods.^13–15^ Eighteen food groups were created and divided into three main groups: healthy, unhealthy foods and animal foods. Healthy food groups involve whole grains, fruits, vegetables, legumes, vegetable oils, nuts, tea and coffee whereas less healthy food groups include sugar-sweetened beverages, refined grains, fruit juices, potatoes, sweets and desserts. Animal food groups comprised animal fats, dairy products, eggs, fish and sea foods, poultry and red meat, and miscellaneous animal-based foods. Table 1 shows the details of food grouping. For each food group, the quintile of consumption was applied and a score of 1 to 5 was regarded to it. For PDI, the scores of 5 and 1 were given to the first and last quintiles of plant food intake, respectively. Furthermore, the participants consuming the highest and lowest quintiles of animal foods received scores of 1 and 5, respectively. Moreover, the scores of 5 and 1 were applied for the highest and lowest quintile of healthy plant foods in order to calculate hPDI, respectively. A score of 1 was an indicator of highest consumption while the score of 5 indicated the lowest intake of unhealthy plant foods and animal food items. Scores were added up so as to calculate PDI and hPDI indexes ranging from 18 to 90. Higher total score for each index was related to higher adherence to it.

**Table 1:** Categorisation of food items comprising 18 food groups for computing plant-based diet Indices (PDI)

Food group	Food item	Total PDI	hPDI
Plant food groups (Healthful)			
Whole grains	Dark breads (eg, barbari, sangak), barley, maize	Positive	Positive
Fruits	Melon, watermelon, honeydew melon, plums, apricot, apples, cherries, sour cherries, peaches, nectarine, pear, fig, date, grapes, kiwi, pomegranate, strawberry, banana, persimmon, berry, oranges, greengage, tangerine, sweet lemon, grapefruits	Positive	Positive
Vegetables	Cauliflower, carrot, tomato, spinach, lettuce, cucumber,eggplant, onion, greens, green bean, green pea, celery, mushroom, pepper, garlic, turnip, pumpkin, courgette	Positive	Positive
Nuts	Almonds, peanut, walnut, pistachio, hazelnut, seeds	Positive	Positive
Legumes	Lentils, split pea, beans, chick pea, fava bean, soy, mung bean	Positive	Positive
Vegetable oils	Olive oil, vegetable oil used for cooking	Positive	Positive
Tea and coffee	Tea, coffee	Positive	Positive
Plant food groups (unhealthful)			
Fruit juices	Apple juice, orange juice, melon juice	Positive	Reverse
Refined grains	Lavash bread, baguette bread, rice, pasta, biscuits, taftun bread	Positive	Reverse
Potatoes	French fries, baked or mashed potatoes, potato or corn chips	Positive	Reverse
Sugar sweetened beverages	Cola, chocolate milk	Positive	Reverse
Sweets and desserts	Cookies, cakes, muffins, pies, chocolates, honey, jam, sugar cubes, sugar, candies, others	Positive	Reverse
Animal food groups			
Animal fat	Butter added to food, butter used for cooking	Reverse	Reverse
Dairy	Low-fat milk, low-fat yoghurt, cheese, Kashk, yoghurt drink, high-fat milk, high-fat yoghurt, cream cheese, cream, ice cream, dripped yoghurt	Reverse	Reverse
Egg	Eggs	Reverse	Reverse
Fish or seafood	Canned tuna, fish	Reverse	Reverse
Meat	Beef and veal, lamb, minced meat, sausage, deli meat, hamburger, chicken, heart, kidney, liver, tongue, brain, offal, rennet	Reverse	Reverse
Misc. animal-based foods	Pizza, mayonnaise	Reverse	Reverse

hPDI, healthful PDI.

### Assessment of other variables

A digital scale (Seca 808, Germany) with an accuracy of 0.1 kg was used to measure weight while participants were with light clothes and unshod. To measure height, unshod, a wallmounted stadiometer with a sensitivity of 0.1 cm (Seca, Germany) was applied. Body mass index (BMI) was computed through dividing weight (in kg) by height (in m^2^). Biomedical factors including white cell count, interleukin-6, C reactive protein, lymphocytes, neutrophils and neutrophil to lymphocyte ratio were collected from individuals’ medical files.

### Statistical analysis

The quantitative variables were described using mean (SD) or median (IQR), separately in the tertiles of the variables healthy and total. Linear regression was used to test if the mean of quantitative variables were different over the tertiles; bootstrapping and robust SEs were used if needed.^16^ A χ^2^ test was used for comparison of categorical variables. Multivariable logistic regression was used to estimate the adjusted ORs per 10 units increase with 95% CIs between the exposure variables hPDI and total PDI with the outcome hospitalisation. The confounders, age, sex, BMI and energy intake were identified from the literature and adjusted for in the analysis. The linearity assumption for the exposure and quantitative confounders was assessed using fractional polynomials.^17 18^ Values of p<0.05 were considered significant.

## Results

General characteristics of participants across tertiles of total PDI and hPDI are presented in table 2. There was no strong evidence against no differences between categories of total PDI and hPDI scores in terms of age, sex, weight, BMI and laboratory markers.

**Table 2:** General characteristics of participants according to tertiles of healthy plant-based diet index (hPDI) and total PDI*

Variable	hPDI score				Total PDI score			
	Tertile 1	Tertile 2	Tertile 3	P value†	Tertile 1	Tertile 2	Tertile 3	P value†
Participants (n)	47	45	49		47	42	52	
Inpatient (%)	30 (63.8)	15 (33.3)	8 (16.3)	<0.001	29 (61.7)	13 (31.0)	11 (21.2)	<0.001
Age (years)	48.97 (13.98)	45.18 (17.89)	44.55 (15.66)	0.34	48.23 (15)	46.29 (17.78)	44.38 (15.15)	0.35
Gender (female)	24 (51%)	23 (51%)	20 (41%)	0.56	24 (51%)	22 (52%)	20 (38%)	0.31
Weight (kg)	77.89 (11.87)	77.62 (14.63)	76.75 (10.77)	0.84	78.10 (12.07)	77.21 (13.91)	76.94 (11.57)	0.92
BMI (kg/m^2^)	27.53 (3.97)	26.98 (4.55)	26.37 (2.84)	0.27	27.53 (4.04)	26.98 (4.31)	26.41 (3.17)	0.39
WCC (10^6^/μL)	6.26 (1.95)	5.95 (2)	6.42 (2.45)	0.57	6.06 (1.94)	5.85 (1.65)	6.69 (2.62)	0.23
Lymphocytes (%)	33.50 (10.65)	30.32 (7.91)	32.09 (7.93)	0.40	33 (10.92)	31.03 (7.95)	31.85 (7.75)	0.68
Neutrophils (%)	55.57 (10.8)	57.35 (10.24)	57.06 (8.63)	0.71	54.8 (10.98)	57.9 (10.14)	57.26 (8.4)	0.52
IL-6 (pg/mL)	2 (2–8.02)	2 (2–3.9)	3.71 (2–8.70)	0.72	2 (2–8.02)	2.46 (2–7.35)	2.27 (2–8.33)	0.71
NLR	2.04 (1.41)	2.27 (1.86)	2.06 (1.35)	0.82	2.05 (1.42)	2.25 (1.9)	2.07 (1.32)	0.86
CRP (U/L)	5.34 (7.1)	5.88 (6.53)	5.19 (6.74)	0.89	5.32 (7.11)	6.12 (6.73)	5.07 (6.55)	0.76

*Data were presented as mean (SD) and median (IQR) except for gender and inpatient rate for which No. (%) were given.

†By the use of linear regression with bootstrapping and χ^2^ test for continuous and categorical variables, respectively.

BMI, body mass index; CRP, C reactive protein; NLR, neutrophil to lymphocyte ratio; WCC, white cell count.

Table 3 indicates multivariable-adjusted OR estimates with 95% CIs, and p values for COVID-19 hospitalisation according to total PDI and hPDI. Fractional polynomials confirmed linearity assumption for both exposures and quantitative confounders. In the crude model, when total PDI and hPDI scores increased by 10 units, the odds of COVID-19 hospitalisation significantly diminished by 10% and 9%, respectively. However, after adjustment for confounders including age, sex, energy intake and BMI, the odds of COVID-19 hospitalisation declined by 58% per 10 units increase in total PDI (OR 0.42; 95% CI 0.22 to 0.80). Each 10 units higher score of hPDI resulted in 55% lower odds of hospitalisation (OR 0.45; 95% CI 0.26 to 0.78).

**Table 3:** Multivariable-adjusted* OR estimates with 95% CIs and p values between total PDI and hPDI with COVID-19 hospitalisation

	Total PDI			Healthy PDI (hPDI)		
	n	Mean	SD	n	Mean	SD
Outpatient	88	43.81	8.33	88	49.1	9.98
Inpatient†	53	37.3	7.09	53	40.81	8.47
	**OR**	**95% CI**	**P value**	**OR**	**95% CI**	**P value**
Model I‡	0.90	0.86 to 0.94	<0.001	0.91	0.87 to 0.95	<0.001
Model II§	0.42	0.22 to 0.80	0.009	0.45	0.26 to 0.78	0.005

*Analysis was adjusted by age, sex, energy intake and body mass index using multivariable logistic regression.

†Not ICU patients.

‡Crude.

§Adjusted.

## Discussion

Although it has been shown that healthy eating pattern has an essential role in boosting the human immune system,^6^ the relationship between PDI and the risk of COVID severity has not yet been elucidated. This study was conducted to evaluate whether plant-based diets are useful to alleviate COVID-19 severity.

In this study, a significant association was observed between PDI and the reduced risk of COVID-19 severity after controlling potential confounders. Based on the findings of the current study, higher scores of PDI were significantly related with a decreased odds of COVID-19 hospitalisation by 59% within fully adjusted model for potential confounders. In other words, we observed that patients in the last tertile of PDI and hPDI had less risk of being hospitalised in comparison to those in the lowest tertile. Our findings are aligned with preliminary evidence showing that the burden of infectious diseases could be reduced by improving nutrition.^6 19–21^ Our findings also concur with the study (Prevencion con ‘Dieta Mediterranea) in which adherence to plant-rich dietary patterns was inversely associated with all-cause mortality.^13^ A review of studies, although scarce, indicates the general benefits of plant-based diet in diminishing the burden of COVID-19.^22^ In accordance with our finding, Merino *et al* revealed that healthy plant-based foods could decrease the risk and severity of COVID-19.^21^ In this large survey, it was shown that as the quality of the diet rises, the risk of disease COVID-19 (HR 0.91) and severe COVID-19 (HR 0.59) diminishes. This association was more pronounced among patients with lower socioeconomic status. Our results could expand previous individual nutrient examinations and highlight the beneficial association of healthy dietary patterns.^23 24^

According to a recent population-based case and control study in six countries, there were significant inverse associations between plant-based diets or pescatarian diets with the odds of moderate-to-severe COVID-19.^8^ Researchers in the study also found that following plant-based diets could protect subjects against severe COVID-19. In this survey, COVID-19 cases and controls completed a web-based questionnaire about demographic characteristics, dietary information and COVID-19 outcomes. The results of this study showed that participants following a plant-based diet had a 73% lower risk of moderate-to-severe COVID-19.

There are several mechanisms involved in lowering the risk of COVID-19 hospitalisation following healthful plant-based diet. One of the possible explanations for the positive correlation between PDI and reducing the risk of hospitalisation due to COVID-19 in this study is the support of the immune system following a healthy diet.^6 25^ Moreover, great amounts of nutrients including phytochemicals, fibre, antioxidants, unsaturated fatty acids, vitamins A, C and E, folate, and mineral are found in plant-based diets.^26 27^ It has been proven that these nutrients could improve the immune system by means of increasing antibody production, proliferation of lymphocytes and reduction of oxidative stress.^6 28^ Reduced risk of respiratory infections including common colds, influenza and pneumonia, as well as reduced severity and duration of illness following supplementation of some of these nutrients, specifically, vitamins A, C, D and E have been reported.^6 29–32^

This study had some limitations. Although we adjusted our analysis for several confounders, some confounders such as physical activity, socioeconomic status and serum vitamin D were not assessed. The inability to confirm a causal relationship between PDI and COVID-severity was another limitation, like any other observational study.

## Conclusion

All in all, following a plant-based diet, could be effective in preventing COVID-19 severity. The findings of this study support our hypothesis that the higher the PDI, the lower the chance of hospitalisation in patients with COVID-19. Future prospective studies are warranted to confirm our findings.

## Data Availability

Data are available on reasonable request.
